# Melatonin: Clinical Perspectives in Neurodegeneration

**DOI:** 10.3389/fendo.2019.00480

**Published:** 2019-07-16

**Authors:** Daniel P. Cardinali

**Affiliations:** Faculty of Medical Sciences, Pontificia Universidad Católica Argentina, Aires, Argentina

**Keywords:** aging, Alzheimer's disease, glymphatic system, melatonin, mild cognitive impairment, neurodegeneration, oxidative stress, Parkinson's disease

## Abstract

Prevention of neurodegenerative diseases is presently a major goal for our Society and melatonin, an unusual phylogenetically conserved molecule present in all aerobic organisms, merits consideration in this respect. Melatonin combines both chronobiotic and cytoprotective properties. As a chronobiotic, melatonin can modify phase and amplitude of biological rhythms. As a cytoprotective molecule, melatonin reverses the low degree inflammatory damage seen in neurodegenerative disorders and aging. Low levels of melatonin in blood characterizes advancing age. In experimental models of Alzheimer's disease (AD) and Parkinson's disease (PD) the neurodegeneration observed is prevented by melatonin. Melatonin also increased removal of toxic proteins by the brain glymphatic system. A limited number of clinical trials endorse melatonin's potentiality in AD and PD, particularly at an early stage of disease. Calculations derived from animal studies indicate cytoprotective melatonin doses in the 40–100 mg/day range. Hence, controlled studies employing melatonin doses in this range are urgently needed. The off-label use of melatonin is discussed.

## Introduction

Symmetrical losses of neurons in the cognitive, motor, or sensory systems characterize neurodegenerative diseases showing prominent cognitive symptoms, like Alzheimer's disease (AD) and frontotemporal dementia, or predominantly motor symptoms like Parkinson's disease (PD), Huntington's disease or amyotrophic lateral sclerosis. Neuronal death in these entities can be ascribed to interrelated processes like free radical-mediated degeneration, mitochondrial dysfunction, low degree of inflammation and excitotoxicity (1, 2). Although the regular intake of antioxidants has been proposed for prevention of neurodegeneration, its effectiveness, however, has been questioned (3). In this context, the cytoprotection given by melatonin use deserves to be considered.

Melatonin, an unusual phylogenetically conserved compound present in all known aerobic phyla, has a promising significance as a cytoprotective molecule in addition to its chronobiotic properties (4). The pineal gland is the demonstrable source of melatonin in circulation, the decrease in plasma melatonin being one of the characteristics of advancing age in humans (5). The focus of this article is on the clinical use of melatonin in neurodegenerative diseases. The discussion of the basic biological data is restricted to its relevance for melatonin doses potentially employable in humans.

## Basic Biology of Melatonin

“Chronobiotics” are defined as drugs displaying the capacity to synchronize or to increase the amplitude of the circadian rhythms, melatonin being the prototype (6, 7). Light-dark variation of melatonin synthesis defines the essential role of melatonin as a chronobiotic (8). Melatonin “opens the doors of sleep” by inhibiting the propensity to wakefulness derived from the suprachiasmatic nuclei (SCN) in late evening (9, 10). On the other hand, melatonin is the chemical code of darkness, an information crucial to the neuroendocrine system (11).

Although in mammals the circulating melatonin derives almost exclusively from the pineal gland (5), the methoxyindole is synthesized locally in most cells, tissues, and organs (12). Indeed, there is now strong evidence that melatonin is produced in every animal cell that has mitochondria (13, 14), melatonin being involved, among other functions, in the elimination of free radicals and the regulation of immune response to achieve cytoprotection (15).

MT_1_ and MT_2_ receptors, all belonging to the superfamily of membrane receptors associated with G proteins (G-protein coupled receptors, GPCR) are involved in the chronobiotic action of melatonin (16). MT_1_ and MT_2_ receptors have been identified in the SCN, hippocampus, thalamus, retina, vestibular nuclei and cerebral and cerebellar cortex (17).

More recently, another member, GPR50, was included in the melatonin receptor subfamily showing high sequence homology with MT_1_ and MT_2_ (18). However, GPR50 does not bind melatonin or any other known ligand. Rather, they form homo- and heteromers between each other and with other GPCRs (19).

Melatonin is not only generated and metabolized in the mitochondria but it was recently claimed that the neuroprotective effects of melatonin on the brain injury induced by ischemia/reperfusion were mediated by MT_1_ receptors located in the mitochondria but not in the membrane (20). This is remarkable because a GPCR like the MT_1_ is known as a cell-surface receptor that transmits extracellular signals into the cell.

Melatonin, an amphiphilic substance, can penetrate cell membranes. In the cytoplasm melatonin interacts with calmodulin and tubulin (21). Melatonin also enters the cell nucleus where the receptor sites were supposed to belong to the orphan receptor superfamily RZR/ROR (15). However, RZR/ROR demonstrably does not bind melatonin. Rather, melatonin may act indirectly via this transcription factor, e.g., by affecting the circadian accessory oscillator component RORα through sirtuin-1 (SIRT-1) activation (22).

The cytoprotective activity of melatonin exceeds that mediated via receptors. The amounts of melatonin found in almost every cell are much higher than those in circulation (12). Although the capacity of mitochondria to synthesize melatonin is now confirmed, intracellular melatonin does not get the extracellular space. Indeed, the doses of melatonin needed to change intracellular melatonin concentration are much higher than those employed as a chronobiotic (23, 24).

In cell cultures, physiologically relevant effects of melatonin are revealed at doses in the range of 10^−8^ to 10^−9^ M, these concentrations being enough for almost complete or total receptor saturation (25, 26). However, most studies on neuroprotective and anti-inflammatory effects in animals employ pharmacological doses, which clearly exceed the saturation of the receptor.

The focus in this review is on melatonin effects on neurodegeneration in animal studies as related to the possible human doses to be employed. It must be noted that cell line studies regarding AD and melatonin have delineated important melatonin mediated mechanisms in the line of prevention against AD as well. A comprehensive review on melatonin activity to reverse disrupted signaling mechanisms in neurodegeneration, including proteostasis dysfunction, disruption of autophagic integrity, and anomalies in the insulin, Notch, and Wnt/β-catenin signaling pathways has just been published (27).

In a way largely independent of receptors melatonin has antioxidant and scavenging effects (28). Melatonin has intrinsic free radical scavenging activity as well as is metabolized to compounds that display a higher antioxidant capacity. In addition, melatonin inhibits the synthesis of prooxidant enzymes and facilitates that of antioxidant enzymes. Melatonin exceeds that capacity of vitamin C and E to protect from oxidative damage (29). Melatonin also exerts cytoprotection in ischemia (independently of free radical scavenging) presumably via mitochondrial membrane stabilization (24).

Immunomodulation by melatonin includes proinflammatory and anti-inflammatory effects (30–32). The anti-inflammatory actions are of great medical interest since they are found in high-grade inflammation like brain injury, ischemia/reperfusion or sepsis, as well as in low-grade inflammation like aging or neurodegenerative processes.

The anti-inflammatory properties of melatonin are exerted by inhibiting the binding of nuclear factor κB (NF κB) to DNA (thus decreasing the synthesis of proinflammatory signals), by inhibiting cyclooxygenase (Cox) (21) mainly Cox-2 (33), and by suppressing the expression of the inducible nitric oxide synthase (iNOS) (34). Other signaling pathways involved include prevention of inflammasome NLRP3 activation, upregulation of nuclear factor erythroid 2-related factor 2 and inhibition of toll-like receptor-4 activation and high-mobility group box-1 signaling. The upregulation of SIRT-1 by melatonin appears to be of major importance. Collectively, these effects of melatonin are reflected in reduced levels of proinflammatory cytokines and increased production of anti-inflammatory cytokines (31).

The γ-aminobutyric acid (GABA)-ergic system can be involved in the neuroprotection mediated by melatonin. Indeed, melatonin exerts anti-excitatory and sedative effects (35, 36) and information exists that melatonin gives protection to neurons from the toxicity of the amyloid-β (Aβ) peptide via activation of GABAergic receptors (37). The up-regulation of GABA activity by melatonin could not be blocked by the melatonin receptor antagonist luzindole but it was impaired by the benzodiazepine antagonist flumazenil, suggesting an allosteric modulation of GABA_A_ receptors by melatonin (38).

Melatonin displays also anti-excitotoxic activity. For example, melatonin prevents neuronal death induced by kainate, an ionotropic glutamate receptor agonist (39), and its administration protects hippocampal CA1 neurons from transient anterior ischemia (40), or from high doses of glucocorticoids (41). The lack of effects of luzindole or of the MT_2_ antagonist 4-phenyl-2-propionamidotetralin (4-P-PDOT) excludes the participation of melatonin receptors in melatonin anti-excitotoxic activity (42).

In addition to the animal models of AD and PD that are discussed in the present paper, melatonin has been shown to reduce neuronal damage due to the toxicity of cadmium (43, 44), hyperbaric hyperoxia (45, 46), toxicity by δ-aminolevulinic acid (47), γ radiation (48), focal ischemia (49), brain trauma (50, 51), and that resultant from several neurotoxins (52).

## Melatonin Activity in Animal Models of AD

The extracellular deposits of Aß-formed senile plaques and the intracellular accumulation of neurofibrillary tangles due to hyperphosphorylation of tau protein are the pathological signatures of AD (1, 2). Aß promotes neuronal degeneration in AD neurons that have become vulnerable to age-related increases of oxidative stress and altered cellular energy metabolism. Hyperphosphorylated tau protein promotes the assembly of microtubules and is an important factor in stabilizing microtubules (1, 2).

The 39–43 amino acid residue Aß derives from the amyloid precursor protein (APP). Melatonin interferes with the maturation of APP in several cell lines (53).

Table 1 summarizes the effect of melatonin in transgenic models of AD. The data indicate that melatonin modulates APP and Aß metabolism principally at the initial phases of the pathological process. From the doses of melatonin used in these different transgenic models, the human equivalent dose of melatonin for a 75 kg adult can be calculated by normalization of body surface area (54). Noteworthy, theoretical human equivalent doses calculated from Table 1 results ranged from 2- to 3-orders of magnitude greater than those employed in humans. However, a note of caution must be made since these studies include changes in the expression of genes with mutations characteristic of the hereditary form of AD, only responsible for 5% of AD cases. Senescence-accelerated OXYS rats appears to be a suitable non-transgenic model of sporadic AD (which accounts for 95% of AD patients) as characterized by the progressive age-related aggregation of Aβ and hyperphosphorylation of τ protein as well as mitochondrial dysfunction, loss of synapses, neuronal death, and concomitant cognitive decline (72). Remarkable, a very low dose of melatonin (0.04 mg daily p.o.) was effective to prevent all these changes (73). Additional studies are needed to solve the dose incongruences observed.

**Table 1:** Effect of melatonin on transgenic models of AD.

**References**	**Design**	**Results**	**Melatonin human equivalent dose for a 75 kg adult**
Matsubara et al. (55)	4-month-old APP 695 transgenic mice received 50 mg/kg of melatonin in drinking water for 8, 9.5, 11, and 15.5 months	The administration of melatonin partially inhibited the expected time-dependent elevation of β-amyloid, reduced abnormal nitration of proteins, and increased survival	300 mg/day
Feng et al. (56)	4-month-old APP 695 transgenic mice received 10 mg/kg of melatonin in drinking water for 4 months	Melatonin counteracted learning and memory impairment in transgenic mice, as shown by step-down and step-through passive avoidance tests. Additionally, the decrease in choline acetyltransferase activity in the frontal cortex and hippocampus of transgenic mice was prevented by melatonin	60 mg/day
Quinn et al. (57)	14-month-old transgenic (Tg 2576) mice received 3.6 mg/kg melatonin in drinking water for 4 months	There were no differences between untreated and melatonin-treated transgenic mice in cortical levels Aβ, nor in brain levels of lipid peroxidation product. Melatonin fails to produce antiamyloid or antioxidant effects when initiated after the age of amyloid plaque deposition	20 mg/day
Feng et al. (58)	4-month-old APP 695 transgenic mice received 10 mg/kg of melatonin in drinking water for 4 months	Melatonin prevented the increase of brain thiobarbituric acid reactive substances, the decrease in glutathione content, and the upregulation of the apoptotic-related factors in transgenic mice	60 mg/day
Garcia et al. (59)	5-month-old female transgenic (Tg2576) were exposed for 6 months to aluminum (1 mg/g) or melatonin (10 mg/kg/day in drinking water)	No effect of aluminum on general motor activity was found. A lower habituation pattern was observed in melatonin-treated animals. Aluminum-treated Tg2576 mice showed impaired learning, an effect unmodified by melatonin treatment	60 mg/day
Olcese et al. (60)	Melatonin (20 mg/kg in drinking water) was given to 2–2.5 month-old APP/PS1 transgenic mice for 5 months	Transgenic mice given melatonin were protected from cognitive impairment in working memory, spatial reference learning/memory, and basic mnemonic function. Immunoreactive Aβ deposition was reduced in hippocampus and entorhinal cortex of melatonin treated transgenic mice. Melatonin decreased tumor necrosis factor-α in hippocampus and normalized cortical mRNA expression of antioxidant enzymes	120 mg/day
Garcia et al. (61)	5-month-old female transgenic (Tg2576) were exposed for 6 months to aluminum (1 mg Al/g diet) or melatonin (10 mg/kg/day in drinking water)	The prooxidant effect of aluminum in the hippocampus was prevented by melatonin	60 mg/day
Spuch et al. (62)	9-month-old male APP/PS1 transgenic mice were used. The tacrine–melatonin hybrid (2 μl per mouse, 50 μg/ml) was stereotaxically injected in each lateral ventricle and the animals were killed 6 weeks later	The intracerebral administration of tacrine-melatonin hybrid decreased Aβ-induced cell death and amyloid burden in the brain parenchyma of APP/Ps1 mice. The reduction in Aβ pathology was accompanied by the recovery of cognitive function	–
Bedrosian et al. (63)	Melatonin (1 mg/kg) was given nightly for 4 week to 9-month-old transgenic amyloid precursor protein (APPSWE) mice	A temporal pattern of anxiety-like behavior emerged in elderly mice and in transgenic APP mice i.e., elevated locomotor activity relative to adult mice near the end of the dark phase, and time-dependent changes in basal forebrain acetylcholinesterase expression. Melatonin treatment did not affect the modifications found in elderly or transgenic mice	6 mg/day
Dragicevic et al. (64)	18 to 20-month-old APP/PS1 transgenic mice received 20 mg/kg melatonin in drinking water for 1 month	Melatonin treatment decreased mitochondrial Aβ levels in several brain regions. This was accompanied by a near complete restoration of mitochondrial respiratory rates, membrane potential, and ATP levels in isolated mitochondria from the hippocampus, cortex, or striatum	120 mg/day
Baño et al. (65)	3.5 to 5.5-month-old APP/PS1 double transgenic mouse were given melatonin (5 mg/kg) or ramelteon (2 mg/kg) in drinking water or in re-pelleted food, respectively, for 5.5 months	Many of the circadian and behavioral parameters measured, including hippocampal oxidative stress markers, were not significantly affected in transgenic mice. Whereas, melatonin maintained τ at 24 h for body temperature and locomotor activity, ramelteon treatment had no effect. Brain tissue analysis revealed a significant reduction in hippocampal protein oxidation in transgenic mice treated with melatonin or ramelteon	30 mg/day
Dragicevic et al. (66)	11 to 12-month-old APPsw mice received 100 mg/kg of melatonin for 1 month	Melatonin treatment yielded a near complete restoration of brain mitochondrial function in assays of respiratory rate, membrane potential, reactive oxygen species production, and ATP levels	600 mg/day
Garcia-Mesa et al. (67)	6-month-old 3xTg-AD mice received 10 mg/kg for 6 months. Physical exercise was implemented by free access to a running wheel in the housing cage	Both melatonin and physical exercise decreased soluble amyloid β oligomers, whereas only melatonin decreased hyperphosphorylated tau. Both treatments protected against cognitive impairment, brain oxidative stress, and a decrease in mitochondrial DNA. Only the combined treatment of physical exercise plus melatonin was effective against the decrease of mitochondrial complexes	60 mg/day
McKenna et al. (68)	50 mg/kg ramelteon in drinking water was given to B6C3-Tg (APPswe, PSEN1dE9) 85Dbo/J mice for 6 months	Absence of effect of ramelteon on cognitive performance of AD mice (water maze) or Aβ deposits in cerebral cortex or hippocampus	–
Di Paolo et al. (69)	5-month-old female transgenic (Tg2576) were exposed for 14 months to aluminum (1 mg Al/g diet) or melatonin (10 mg/kg/day in drinking water)	Melatonin improved learning and spatial memory in aluminum-exposed transgenic mice	60 mg/day
Gerenu et al. (70)	4-month-old double-transgenic female APP/PS1 mice were administered with a curcumin/melatonin hybrid (Z-CM-I-1) (50 mg/kg) by oral gavage. Animals were treated 5 times per week for 12 consecutive weeks	Z-CM-I-1 decreased the accumulation of Aβ in the hippocampus and cerebral cortex and reduced inflammatory responses and oxidative stress. Z-CM-I-1 also increased expression of synaptic marker proteins PSD95 and synaptophysin and of complexes I, II, and IV of the mitochondria electron transport chain	150 mg/day
Nie et al. (71)	10-month-old triple transgenic mice (3xTg-AD) received melatonin (10 mg/kg/day in drinking water) for 1 month	Melatonin ameliorated anxiety and depression-like behaviors of 3xTg-AD mice. Hippocampal glutathione S-transferase P 1 (an anxiety associated protein) and complexin-1 (a depression associated protein) were significantly modulated by melatonin	60 mg/day

*The human equivalent dose of melatonin for a 75 kg adult is calculated by normalization of body surface area (54)*.

How melatonin inhibits generation of Aβ remains undefined. Melatonin may interact with Aß_40_ and Aß_42_ inhibiting the formation of progressive β-sheet and/or amyloid fibrils (74, 75). Such interaction appears to be independent on melatonin antioxidant properties (74).

Via blockage of formation of secondary sheets, melatonin may facilitate the peptide clearance induced by proteolytic degradation. Since GSK−3 is a common signaling pathway increasing Aß generation and tau hyperphosphorylation, melatonin could regulate APP and tau processing via protein kinase (PK) C activation (76, 77) and inhibition of GSK-3 pathway (78). Free radical are involved in Aß-induced neurotoxicity and cell death, melatonin effectively protecting cells against oxidative damage *in vitro* (79, 80) and *in vivo* (81–83).

In N2a and SH-SY5Y neuroblastoma cells exposed to wortmannin (84), calyculin A (85, 86), or okadaic acid (87–89) melatonin efficiently attenuated tau hyperphosphorylation via protein kinases and phosphatases, as well as antagonizes the oxidative stress given by these agents (90, 91). Regulation of PK A (92), PK C (93), Ca^2+^/calmodulin-dependent kinase II (94), and mitogen-activated protein kinase are other effects of melatonin unrelated to its antioxidant properties (95).

A crucial phenomenon for brain homeostasis is waste products' elimination by the glymphatic system. The term “glymphatic” describes active, lymphatic-like, water exchange movements in the brain extracellular space (ECS) driven by perivascular astrocytes, which contain aquaporin-4 (AQP4) located in their end feet (96). Since the elimination of Aβ peptide is strongly reduced in AQP4^−/−^ mice (97), occurrence of an AQP4-driven glymphatic Aβ clearance seems feasible.

During sleep, the elimination of Aβ peptides increases considerably (98). Thus, the sleep disturbance found as a comorbidity in AD may contribute to the development and progression of the disease via a failure of Aβ clearance. Sleep deprivation disrupted apolipoprotein E clearance from brain ECS (99). That the glymphatic system participates in tau protein clearance was further indicated by the demonstration that AQP4 deficiency augmented the presence of extracellular tau and neuronal tangle formation in a murine model of traumatic brain injury (100).

An important recent observation by Pappolla et al. indicate that the administration of melatonin to AD transgenic mice augments the glymphatic clearance of Aβ (101). Relevant to this, melatonin is known to preserve slow wave sleep in patients (102). Indeed, glymphatic dysfunction has been related to various neurological disease in addition to AD, like stroke or traumatic brain injury (103).

The rise in the expression of proinflammatory cytokines triggered by microglial activation seems to play a role in the pathogenesis of AD (1, 2). Microglial release of proinflammatory cytokines induced by NF kB, Aß, and nitric oxide is effectively halted by melatonin (83). Binding of NF kB to DNA was also inhibited by melatonin (22, 31).

## Clinical Application of Melatonin in AD

Cerebrospinal fluid (CSF) concentration of melatonin decreases even at the preclinical stages of AD (104). Circulating melatonin correlates negatively with neuropsychological evaluation in mild cognitive impairment (MCI) and AD patients (105). The relative deficiency of melatonin could be the cause or a consequence of neurodegeneration. In any event, the loss of melatonin aggravates the disease and causes early circadian disturbance as shown by “sundowning” (106). Sundowning comprises late afternoon or evening symptoms such as agitation, wandering, disorganized thinking, perceptual and emotional disturbances and reductions in attention. Chronotherapeutic interventions, such as timed administration of melatonin and exposure to bright light, relieve sundowning and improved sleep in AD patients (107, 108).

The irregular sleep/wake found in AD is effectively treated by melatonin (Table 2). A significant decrease in sundowning and reduced variability of sleep onset time were found in 7 out of 10 dementia patients with sleep disorders treated with 3 mg melatonin at bedtime for 3 weeks (109).

**Table 2:** Studies including treatment of AD patients with melatonin.

**Subjects**	**Design**	**Study's duration**	**Treatment**	**Measured**	**Results**	**References**
10 demented patients	Open-label study	3 weeks	3 mg melatonin p.o./daily at bed time	Daily logs of sleep and wake quality completed by caretakers	7 out of 10 dementia patients having sleep disorders treated with melatonin showed a significant decrease in sundowning and reduced variability of sleep onset time	(109)
14 AD patients	Open-label study	22–35 months	6–9 mg melatonin p.o./daily at bed time	Daily logs of sleep and wake quality completed by caretakers. Neuro-psychological assessment	Sundowning was no longer detectable in 12 patients and persisted, although attenuated in 2 patients. A significant improvement of sleep quality was found. Lack of progression of the cognitive and behavioral signs of the disease during the time they received melatonin	(110)
Monozygotic twins with AD	Case report	36 months	One of the patients was treated with melatonin 9 mg p.o./daily at bed time	Neuro-psychological assessment. Neuroimaging	Sleep and cognitive function severely impaired in the twin not receiving melatonin as compared to the melatonin-treated twin	(111)
11 AD patients	Open-label study	3 weeks	3 mg melatonin p.o./daily at bed time	Daily logs of sleep and wake quality completed by the nurses	Significant decrease in agitated behaviors in all three shifts; significant decrease in daytime sleepiness	(112)
14 AD patients	Open-label, placebo-controlled trial	4 weeks	6 mg melatonin p.o./daily at bed time or placebo	Daily logs of sleep and wake quality completed by caretakers. Actigraphy	AD patients receiving melatonin showed a significantly reduced percentage of nighttime activity compared to a placebo group	(113)
25 AD patients	Randomized double blind placebo controlled cross over study	7 weeks	6 mg of slow release melatonin p.o. or placebo at bed time	Actigraphy	Melatonin had no effect on median total time asleep, number of awakenings or sleep efficiency	(114)
45 AD patients	Open-label study	4 months	6–9 mg melatonin p.o./daily at bed time	Daily logs of sleep and wake quality completed by caretakers. Neuro-psychological assessment	Melatonin improved sleep and suppressed sundowning, an effect seen regardless of the concomitant medication employed	(107)
157 AD patients	Randomized placebo-controlled clinical trial	2 months	2.5-mg slow-release melatonin, or 10-mg melatonin or placebo at bed time	Actigraphy. Caregiver ratings of sleep quality	Non-significant trends for increased nocturnal total sleep time and decreased wake after sleep onset were observed in the melatonin groups relative to placebo. On subjective measures, caregiver ratings of sleep quality showed a significant improvement in the 2.5-mg sustained-release melatonin group relative to placebo	(115)
20 AD patients	Double-blind, placebo-controlled study	4 weeks	Placebo or 3 mg melatonin p.o./daily at bed time	Actigraphy. Neuro-psychological assessment	Melatonin significantly prolonged the sleep time and decreased activity in the night. Cognitive function was improved by melatonin	(116)
7 AD patients	Open-label study	3 weeks	3 mg melatonin p.o./daily at bed time	Actigraphy. Neuro-psychological assessment	Complete remission of day night rhythm disturbances or sundowning was seen in 4 patients, with partial remission in other 2	(117)
17 AD patients	Randomizedplacebo-controlled study	2 weeks	3 mg melatonin p.o./daily at bed time (7 patients). Placebo (10 patients)	Actigraphy. Neuro-psychological assessment	In melatonin-treated group, actigraphic nocturnal activity and agitation showed significant reductions compared to baseline	(118)
68-year-old man with AD who developed rapid eye movement (REM) sleep behavior disorder	Case report	20 months	5–10 mg melatonin p.o./daily at bed time	Polysomnography	Melatonin was effective to suppress REM sleep behavior disorder	(119)
50 AD patients	Randomizedplacebo-controlled study	10 weeks	Morning light exposure (2,500 lux, 1 h) and 5 mg melatonin (*n* = 16) or placebo (*n* = 17) in the evening. Control subjects (*n* = 17) received usual indoor light (150–200 lux)	Nighttime sleep variables, day sleep time, day activity, day: night sleep ratio, and rest-activity parameters were determined using actigraphy	Light treatment alone did not improve nighttime sleep, daytime wake, or rest-activity rhythm. Light treatment plus melatonin increased daytime wake time and activity levels and strengthened the rest-activity rhythm	(120)
41 AD patients	Randomizedplacebo-controlled study	10 days	Melatonin (8.5 mg immediate release and 1.5 mg sustained release) (*N* = 24) or placebo (*N* = 17) administered at 10:00 p.m.	Actigraphy	There were no significant effects of melatonin, compared with placebo, on sleep, circadian rhythms, or agitation	(121)

In another study including 14 AD patients treated with 6–9 mg/day for a period of 2–3 years, sleep quality improved (110). Sundowning was no longer detectable except for 2 patients. We also observed improvement of cognitive performance and reduction of amnesia after melatonin treatment. In a case report of monozygotic twins with AD followed for 36 months we reported a better sleep and cognitive function in the twin receiving melatonin (111).

The effectiveness of melatonin to improve sleep and alleviate sundowning were reported in open-label and placebo-controlled studies in AD patients (107, 112, 113, 115–120). Negative results were also published in fully developed AD patients treated with melatonin (114, 121). Indeed, large interindividual differences in sleep and agitation are common among AD patients.

A review of published results on the use of melatonin in AD (122) yielded seven reports (5 open studies, 2 case reports) (*N* = 89 patients) that supported a possible efficacy of melatonin in improving sleep, decreasing sundowning and improving cognitive deterioration. In six double blind, randomized placebo-controlled trials (*N* = 210 patients) sleep quality increased, sundowning decreased, and cognitive performance improved in 4 studies (*N* = 143) whereas there was absence of significant effects in 2 studies (*N* = 67) (122). Two meta-analyses supported the view that melatonin therapy is effective in improving sleep in patients with dementia (123, 124). In addition, the melatonergic agent ramelteon was effective in treating delirium of elderly patients in intensive care units (125).

Whether melatonin has any value in the treatment of fully developed AD remains undefined. It should be noted that the heterogeneity in pathology of the group examined is probably very high at this stage of disease. Therefore, information obtained at an earlier phase could be more valuable.

Patients with MCI have a deficit in cognitive functions with preservation of daily activities. MCI is a clinically important stage to identify and treat people at risk (126) because the estimate of the annual rate of conversion of MCI to dementia can be as high as 10–15%, In fact, the degenerative process in the brain of AD begins 20–30 years before the clinical onset of the disease (127–131).

As shown in Table 3, data published from MCI patients consistently showed that melatonin administration improves cognitive performance and sleep quality. For example, we reported a significant improvement of cognitive and depressive symptoms and sleep quality in 35 patients with MCI treated for up to 2 years with 3–9 mg/day of melatonin as an adjuvant (130). Significantly lower scores in Beck Depression Inventory and better performance in neuropsychological tests and in sleep and wakefulness subjective assessment were documented in 61 outpatients diagnosed with MCI and receiving 3–24 mg of melatonin daily for 15–60 months (134). Collectively, the results of Table 3 indicate that melatonin is an adjuvant drug useful for the treatment of MCI in a clinical setting.

**Table 3:** Studies including treatment of MCI patients with melatonin.

**Subjects**	**Design**	**Study's duration**	**Treatment**	**Measured**	**Results**	**References**
10 patients with MCI	Double-blind, placebo-controlled, crossover study	10 days	6 mg melatonin p.o./daily at bed time	Actigraphy. Neuropsychological assessment	Melatonin enhanced the rest-activity rhythm and improved sleep quality. Total sleep time unaffected. The ability to remember previously learned items improved along with a significant reduction in depressed mood	(132)
26 individuals with age-related MCI	Double-blind, placebo-controlled pilot study	4 weeks	1 mg melatonin p.o. or placebo at bed time	Sleep questionnaire and a battery of cognitive tests at baseline and at 4 weeks	Melatonin administration improved reported morning “restedness” and sleep latency after nocturnal awakening. It also improved scores on the California Verbal Learning Test-interference subtest	(133)
354 individuals with age-related MCI	Randomized, double blind, placebo-controlled study	3 weeks	Prolonged release melatonin (Circadin, 2 mg) or placebo, 2 h before bedtime	Leeds Sleep Evaluation and Pittsburgh Sleep Questionnaires, Clinical Global Improvement scale score and quality of life	PR-melatonin resulted in significant and clinically meaningful improvements in sleep quality, morning alertness, sleep onset latency and quality of life	(134)
60 MCI outpatients	Open-label, retrospective study	9–24 months	35 patients received daily 3–9 mg of a fast-release melatonin preparation p.o. at bedtime. Melatonin was given in addition to the standard medication	Daily logs of sleep and wake quality. Initial and final neuropsychological assessment	Abnormally high Beck Depression Inventory scores decreased in melatonin-treated patients, concomitantly with an improvement in wakefulness and sleep quality. Patients treated with melatonin showed significantly better performance in neuropsychological assessment	(135)
189 individuals with age-related cognitive decay	Long-term, double-blind, placebo-controlled, 2 × 2 factorial randomized study	1–3.5 years	Long-term daily treatment with whole-day bright (1,000 lux) or dim (300 lux) light. Evenin*g* melatonin (2.5 mg) or placebo administration	Standardized scales for cognitive and non-cognitive symptoms, limitations of activities of daily living, and adverse effects assessed every 6 months	Light attenuated cognitive deterioration and ameliorated depressive symptoms. Melatonin shortened sleep onset latency and increased sleep duration but adversely affected scores for depression. The combined treatment of bright light plus melatonin showed the best effects	(108)
22 individuals with age-related cognitive decay	Prospective, randomized, double-blind, placebo-controlled, study	2 months	Participants received 2 months of melatonin (5 mg p.o. /day) and 2 months of placebo	Sleep disorders were evaluated with the Northside Hospital Sleep Medicine Institute (NHSMI) test. Behavioral disorders were evaluated with the Yesavage Geriatric Depression Scale and Goldberg Anxiety Scale	Melatonin treatment significantly improved sleep quality scores. Depression also improved significantly after melatonin administration	(136)
25 MCI outpatients	Randomized, double-blind, placebo-controlled study	12 weeks	11 patients received an oily emulsion of docosahexaenoic acid-phospholipids containing melatonin (10 mg) and tryptophan (190 mg)	Initial and final neuropsychological assessment of orientation and cognitive functions, short-term and long-term memory, attentional abilities, executive functions, visuo-constructional and visuo-spatial abilities, language, and mood	Older adults with MCI had significant improvements in several measures of cognitive function when supplemented with an oily emulsion of DHA-phospholipids containing melatonin and tryptophan for 12 weeks, compared with the placebo. The antioxidant capacity of erythrocytes and membrane lipid composition improved after treatment	(137, 138)
96 MCI outpatients	Open-label, retrospective study	15–60 months	61 patients received daily 3–24 mg of a fast-release melatonin preparation p.o. at bedtime. Melatonin was given in addition to the standard medication	Daily logs of sleep and wake quality. Initial and final neuropsychological assessment	Abnormally high Beck Depression Inventory scores decreased in melatonin-treated patients, concomitantly with an improvement in wakefulness and sleep quality. Patients treated with melatonin showed significantly better performance in neuropsychological assessment. Only 6 out of 61 patients treated with melatonin needed concomitant benzodiazepine treatment vs. 22 out of 35 MCI patients not receiving melatonin	(139)
80 patients diagnosed with mild to moderate AD, with and without insomnia comorbidity, and receiving standard therapy (acetylcholinesterase inhibitors with or without memantine)	Randomized, double-blind, parallel-group study	28 weeks	Patients were treated for 2 weeks with placebo and then randomized (1:1) to receive 2 mg of prolonged release melatonin or placebo nightly for 24 weeks, followed by 2 weeks placebo	The AD Assessment Scale-Cognition (ADAS-Cog), Instrumental Activities of Daily Living (IADL), Mini-Mental State Examination (MMSE), sleep, as assessed by the Pittsburgh Sleep Quality Index (PSQI) and a daily sleep diary, and safety parameters were measured	Patients treated with melatonin had significantly better cognitive performance than those treated with placebo. Sleep efficiency, as measured by the PSQI, component 4, was also better. Differences were more significant at longer treatment duration	(140)
142 patients meeting DSM-IV-TR criteria for major depression disorder were enrolled	Double-blind, placebo-controlled, randomized trial	6 weeks	Combination treatment: (buspirone 15 mg with melatonin- 3 mg) vs. buspirone 15 mgmonotherapy, vs. placebo	Clinical global impression of severity (CGI-S) and improvement (CGI-I), the QIDS-SR16, and the Hamilton rating scale for anxiety (Ham-A) at the baseline, week 2, week 4, and week 6 endpoint	Treatment responders improved significantly more on the total CPFQ than non-responders regardless of treatment assignment. The cognitive dimension of the CPFQ score favored the combination treatment over the other two groups	(141)
139 patients older than 65 year. of age scheduled for hip arthroplasty	Prospective cohort study	7 days	Patients were randomized to receive 1 mg oral melatonin or placebo daily 1 h before bedtime 1 day before surgery and for another 5 consecutive days post-operatively	Subject assessment, including Mini-Mental State Examination (MMSE) score, subjective sleep quality, general well-being, post-operative fatigue, and visual analog scale for pain were evaluated pre-operatively and at days 1, 3, 5, and 7 after surgery	The MMSE score in the control group decreased significantly after surgery. The MMSE score in the melatonin group remained unchanged during the 7 days of monitoring. In addition, significant post-operative impairments of subjective sleep quality, general well-being, and fatigue were found in the control group when compared with the melatonin group	(142)

The mechanisms that explain the therapeutic effect of melatonin in patients with MCI have not yet been defined. Promotion of slow-wave sleep in the elderly could be beneficial in MCI by increasing the functioning of the glymphatic system, or the secretion of growth hormone and neurotrophins, linked to the restorative phase of sleep.

The question of whether melatonin has a therapeutic value in the prevention or treatment of MCI deserves further analysis. Multicenter double-blind studies are needed to explore and further investigate the potential and utility of melatonin as a preventive drug against dementia. The doses of melatonin used should be re-evaluated in view of the equivalent human doses of melatonin derived from preclinical data, as indicated in Table 1. Unfortunately, of the 64 clinical trials related to melatonin in an initial state (recruitment and non-recruitment) listed in PubMed (ClinicalTrials.gov Search results 01/03/2019) none is directed to this query.

## Studies on Melatonin Activity in Animal Models of PD

The progressive degeneration of neurons containing dopamine (DA) in the substantia nigra pars compacta (SNpc) characterizes PD (143). Since Lewy bodies are found not only in DA neurons but also in noradrenergic neurons of the brainstem, in serotonergic neurons of the raphe nuclei and in specific cholinergic neurons, PD is seen as a progressive disease affecting a variety of neurotransmitter systems. This explains the number of non-motor symptoms in PD, such as genitourinary, gastrointestinal, respiratory and cardiovascular disorders, anosmia and neuropsychiatric, visual, and sleep-related disorders. In fact, the non-motor preclinical phase of PD can cover more than 20 years, the relevance of neuroprotection being evident in this respect (144).

The inflammatory signature found in the pathogenesis of PD includes microglial activation, astrogliosis and lymphocytic infiltration (145). Several inflammatory mediators, e.g., NF-κB, interleukin (IL)-1, IL-6, Cox-2, tumor necrosis factor-α, iNOS, and interferon-γ are produced by glial cells (2).

PD and other Lewy body diseases are characterized by the aggregation of fibrillar α-synuclein (146). Mitochondrial dysfunction plays a role in this process since the folding and aggregation of proteins are promoted by free radicals (147, 148).

To develop animal models of altered brain DA function, 6-hydroxydopamine (6-OHDA), or the neurotoxin 1-methyl-4-phenyl-1,2,3,6 tetrahydropyridine (MPTP), were injected into the nigrostriatal pathway of the rat (149).

Because of its potentiality to cause the disease in humans and in subhuman primates, MPTP is preferred among other neurotoxins to emulate parkinsonism in animal models. MPTP is selectively taken up by astrocytes and is metabolized into methyl 1-4 phenyl pyridinium (MPP^+^), this cation causing increased production of free radicals, depletion of ATP, and apoptosis. MPTP toxicity is selective to SNpc neurons and induced loss of striatal spines in non-human primates (150). Such striatal spine loss is a consistent neuropathologic finding in post-mortem PD human brains. Although the MPTP-treated monkey is considered the best experimental model of PD, a major drawback is the consistent lack of other neuronal loss besides the nigrostriatal dopaminergic system (151).

In Table 4 the *in vivo* effects of melatonin in several experimental models of PD are shown. Most experiments support the role of melatonin in prevention and treatment of experimental PD. As in the case of Table 1, the human equivalent doses of melatonin for a 75 kg adult, calculated by normalization of body surface area (54), are quoted for the sake of comparison with those employed in PD patients. Again, theoretical human equivalent doses derived from animal data are considerably greater than those employed in humans.

**Table 4:** *In vivo* effect of melatonin in animal models of PD.

**References**	**Design**	**Results**	**Melatonin human equivalent dose for a 75 kg adult**
Burton et al. (152)	Wistar rats receiving 6-OHDA injection into the SNc were treated with melatonin (1 and 10 mg/kg, i.p.)	Melatonin treatment inhibited apomorphine-induced rotational behavior	12 and 120 mg
Acuña-Castroviejo et al. (153)	C57BL/6 mice receiving an injection of MPP^+^ were treated with melatonin (10 mg/kg, i.p.)	Melatonin treatment prevented MPTP-induced lipid peroxidation and TH-positive neuronal loss in striatum	60 mg
Jin et al. (154)	Sprague-Dawley rats receiving an injection of MPP^+^ into the SNc were treated with melatonin (10 mg/kg, i.p.)	Melatonin treatment reduced lipid peroxidation and protected against DA neuronal loss induced by MPP^+^	120 mg
Joo et al. (155)	Sprague-Dawley rats receiving 6-OHDA injections into the striatum were administered with melatonin (3 and 10 mg/kg, i.p.)	Melatonin treatment counteracted the 6-OHDA-induced changes in striatal DA synthesis and levels	36 and 120 mg
Kim et al. (156)	Sprague-Dawley rats receiving 6-OHDA injections into the striatum were treated with melatonin (3 or 10 mg/kg, i.p.)	Melatonin treatment reduced motor deficit and protected against 6-OHDA-induced loss of dopaminergic neurons	36 and 120 mg
Dabbeni-Sala et al. (157)	Sprague-Dawley rats receiving 6-OHDA injection into the SNc were treated with melatonin (50 ± 7.5 μg/h, s.c.)	Melatonin treatment prevented apomorphine-induced rotational behavior and mitochondrial damage	15 mg
Aguiar et al. (158)	Wistar rats receiving 6-OHDA injections into the striatum were administered with melatonin (2, 5, 10, and 25 mg/kg, i.p.)	Melatonin treatment prevented apomorphine-induced rotational behavior and depletion of striatal DA and serotonin levels	24–300 mg
Chen et al. (159)	Wistar rats receiving an injection of MPP^+^ were treated with melatonin (10 mg/kg, i.p.)	Melatonin decreased MPP^+^-induced toxicity and recovered GSH levels	120 mg
Khaldy et al. (160)	C57BL/6 mice receiving an injection of MPP^+^ were treated with melatonin (5 or 10 mg/kg i.p.)	Melatonin protected damage of mitochondrial complex I activity in nigrostriatal neurons	30 and 60 mg
Sharma et al. (161)	Sprague-Dawley rats receiving 6-OHDA injections into the striatum were treated with melatonin (4 μg/mL) in drinking water	Melatonin normalized motor deficits and augmented TH immunoreactivity	6 mg
Singh et al. (162)	Sprague-Dawley rats receiving 6-OHDA injections into the striatum were treated with melatonin (0.5 mg/kg, i.p.)	Melatonin treatment prevented apomorphine-induced rotational behavior	6 mg
Saravanan et al. (163)	Sprague-Dawley rats were injected with rotenone into the SN. Melatonin (10, 20, or 30 mg/kg) was administrated i.p.	Melatonin reduced the levels of hydroxyl radicals in mitochondria and protected GSH levels and antioxidant enzymes activities in SN	120, 240, and 360 mg
Huang et al. (164)	Wistar rats receiving an injection of MPP^+^ were treated with melatonin (10 mg/kg, i.p.)	Melatonin protected DA neurons from apoptosis induced by MPP^+^	120 mg
Tapias et al. (165)	C57BL/6 mice received a single injection of MPTP. Melatonin (20 mg/kg) was given s.c.	Melatonin treatment prevented the MPTP-induced mitochondrial increase of NO, inhibited lipid peroxidation and protected complex I activity in striatum and SNc	120 mg
Patki et al. (166)	C57BL/6 mice received MPTP i.p. injections for 5 weeks. Melatonin (5 mg/kg) was administered i.p.	Melatonin protected against MPTP-induced DA neurons loss and locomotor activity deficit, and recovered mitochondrial respiration, ATP production, and antioxidant enzyme levels in SNc	30 mg
Singhal et al. (167)	Swiss mice treated with maneb plus paraquat received melatonin (30 mg/kg/day, i.p.)	Melatonin treatment protected lipid peroxidation and TH-positive neurons degeneration and prevented apoptosis	180 mg
Gutierrez-Valdez et al. (168)	Wistar rats receiving 6-OHDA injections into the medial forebrain bundle were treated with melatonin (10 mg/kg, p.o.)	Melatonin treatment improved motor performance without causing dyskinesia. Melatonin also protected TH-positive neurons and neuronal ultrastructure of striatum	120 mg
Brito-Armas et al. (169)	Sprague-Dawley rats injected with lentiviral vectors encoding mutant human α-synuclein in the SNc received melatonin treatment (10 mg/kg/day, i.p.)	Melatonin treatment prevented the loss of TH-positive neurons	120 mg
Zaitone et al. (170)	Swiss mice received 4 injections of MPTP. Melatonin was given p.o. (5 or 10 mg/kg/day)	Melatonin treatment recovered motor performance, striatal DA level, GSH, and antioxidant enzyme activities, and reduced lipid peroxidation. Melatonin improved the motor response to L-DOPA	30 and 60 mg
Bassani et al. (171)	Wistar rats were i.p. injected with rotenone. Melatonin (10 mg/kg) was administrated i.p.	Melatonin treatment protect TH-positive neurons in SNc and striatal levels of DA	120 mg
Yildirim et al. (172)	Wistar rats receiving 6-OHDA injections into the medial forebrain bundle were treated with melatonin (10 mg/kg, i.p.)	Melatonin prevented oxidative damage and apoptosis of dopaminergic neurons	120 mg
Naskar et al. (173)	BALB/c mice treated with MPTP received melatonin (10, 20, or 30 mg/kg, i.p.)	Melatonin protected against MPTP-induced TH-positive neurons loss in SNc and enhanced the therapeutic efect of L-DOPA	60, 120, and 180 mg
Ozsoy et al. (174)	Wistar rats receiving 6-OHDA injections into the medial forebrain bundle were treated with melatonin (10 mg/kg/day, i.p.)	Melatonin treatment protected DA neurons against changes in antioxidant enzyme activities and lipid peroxidation	120 mg
Carriere et al. (175)	Sprague Dawley rats were injected with rotenone. Melatonin (4.0 μg/mL) was given in drinking water	Melatonin treatment protected motor deficit and loss of TH-positive neurons in striatum and SNc after rotenone	6 mg
Li et al. (176)	Wistar rats were injected with 6-OHDA in the SNc and ventral tegmental area and received i.p. injections of melatonin (5 mg/kg)	Melatonin prevented DA neuronal damage	60 mg
Lopez et al. (177)	C57BL/6 mice receiving MPTP were administered melatonin (10 mg/kg s.c.)	Melatonin administration prevented the disruption of mitochondrial oxygen consumption, increased NOS activity and reduced locomotor activity induced by MPTP, independently of its anti-inflammatory properties	60 mg
Paul et al. (178)	Wistar rats injected with homocysteine in the SNc received melatonin treatment (10, 20, or 30 mg/kg/day, i.p.)	Treatment of melatonin protected against nigral DA loss and improved mitochondrial complex-I activity in SN	120, 240, and 360 mg

*The human equivalent dose of melatonin for a 75 kg adult are calculated by normalization of body surface area (54)*.

Most of the studies summarized in Table 4 include pretreatment with the methoxyindole and therefore they rely on the neuroprotective effect of melatonin preventing the death of dopaminergic neurons and consequently motor dysfunction (Table 4). In addition, some studies failed to observe motor benefits of melatonin treatment in animal models of PD regardless of protection of neurodegeneration. For example, Bassani et al. reported that the melatonin post-treatment for 28 days preserved tyrosine hydroxylase positive neurons and DA levels of rotenone-lesioned rats and improved the depressive-like behavior in the absence of significant improvement of motor deficit (171).

In PD patients, administration of 3 mg of melatonin for 4 weeks improved the quality of sleep but did not affect motor symptoms (179). Moreover, by evaluating the effects of slow release melatonin preparation via intracerebroventricular implants in rats injected with 6-OHDA or MPTP, Willis and Armstrong (180) reported that melatonin indeed deteriorated motor performance. Remarkably, other studies reported an enhancement of motor deficit in animal models of PD after the administration of the antagonist of melatonin receptors ML-23 (181). The beneficial effect of melatonin antagonism on motor symptoms of PD could be explained by inhibition of DA release by the methoxyindole (182).

Regardless of these discrepancies, melatonin preventing activity on PD-related neurodegeneration is generally accepted (183–185). For example, melatonin inhibits α-synuclein assembly and attenuated kainic acid-induced neurotoxicity (186) and arsenite-induced apoptosis (187). Melatonin also impaired the augmented expression of α-synuclein in DA containing neurons following amphetamine administration (188, 189). Melatonin blocked α-synuclein fibril formation and destabilized preformed fibrils by inhibiting protofibril formation and secondary structure transitions and by reducing α-synuclein cytotoxicity (169, 190).

An insufficient clearance by the autophagic–lysosomal network (146) can explain the accumulation and spread of oligomeric forms of neurotoxic α-synuclein. In addition, other clearance pathways are compromised, like the ubiquitin–proteasome system, the autophagy mediated by chaperone, extracellular clearance by proteases, or entrance into the general circulation via the glymphatic system (146).

As above mentioned, the elimination of waste products by the glymphatic system considerably contributes to recovery processes in the brain. The role of AQ4 water channels in the glymphatic system seems to be crucial and, remarkably, AQ4 expression is severely disrupted in PD brains (191). It may explain why the CSF α-synuclein levels inversely correlate with symptoms in PD patients (192). The association of loss of sleep with impairment of the glymphatic clearance is important in the case of PD because rapid eye movement sleep behavior disorder (RBD) is a prodrome of PD. Melatonin administration to animals augments glymphatic clearance (101) as well as preserved sleep in patients. Curiously, melatonin was not listed in the myriad of drugs affecting anomalous protein clearance in the brain (146).

Symptomatically, an effective treatment for PD is the supplementation of DA in its precursor form L-dihydroxyphenylalanine (L-DOPA) that crosses the blood brain barrier. However, long-term administration of -L-DOPA leads to motor side effects like dyskinesias (193, 194). Moreover, administration of L-DOPA in high doses leads to production of neurotoxic molecules like 6-OHDA. Therefore, efforts to reduce the intake or to compensate for the side effects of L-DOPA are in the vogue. In MPTP-treated mice, melatonin, but not L-DOPA, restored striatal spine density, supporting the application of melatonin as an adjuvant to L-DOPA therapy in PD (173).

## Clinical Use of Melatonin in PD

Approximately 3/4 of the dopaminergic cells in the SNpc need to be lost to uncover motor symptomatology in PD. However, non-motor symptoms like hyposmia, depression, or RBD (characterized by the occurrence of vivid, intense, and violent movements during REM sleep) precede the onset of PD for years and are index of worse prognosis (144). Indeed, up to 65% of patients showing RBD developed PD 10–13 years later (195).

Table 5 summarizes the clinical studies reporting melatonin use in PD. Daily administration of 3–12 mg of melatonin at bedtime is effective in the treatment of RBD (198–206). Polysomnography (PSG) in RBD patients treated with melatonin showed significant decreases in number of R epochs without atonia and in movement time during REM sleep, contrasting with the persistence of muscle tone in R sleep seen with patients treated with clonazepam. Based on these data, a clinical consensus recommended melatonin use in RBD at Level B (207).

**Table 5:** Studies including treatment of PD and RBD patients with melatonin.

**Subjects**	**Design**	**Study's duration**	**Treatment**	**Measured**	**Results**	**References**
40 PD patients	Open-label, placebo-controlled trial	2 weeks	5–50 mg melatonin p.o./daily at bed time. All subjects were taking stable doses of antiparkinsonian medications	Actigraphy	Relative to placebo, treatment with 50 mg of melatonin significantly increased night time sleep, as revealed by actigraphy. As compared to 50 mg or placebo, administration of 5 mg of melatonin was associated with significant improvement of sleep in the subjective reports	(196)
18 PD patients	Open-label, placebo-controlled trial	4 weeks	3 mg melatonin p.o./daily at bed time	Polysomnography (PSG). Subjective evaluation by the Pittsburgh Sleep Quality Index and Epworth Sleepiness Scale	On initial assessment, 14 patients showed poor quality sleep EDS. Increased sleep latency (50%), REM sleep without atonia (66%), and reduced sleep efficiency (72%) were found in PSG. Melatonin significantly improved subjective quality of sleep. Motor dysfunction was not improved using melatonin	(179)
38 patients with PD without dementia and with complaints on sleep disorders	Open-label trial	6 weeks	Group 1 (*n* = 20) received 3 mg melatonin in addition to the previous dopaminergic group 2 (*n* = 18) received clonazepam 2 mg at night	Polysomnography (PSG) at baseline and at the end of the trial. Subjective evaluation by the PD sleep scale (PDSS) and the Epworth Sleepiness Scale (ESS). Neuropsychological testing using MMSE, five-word test, digit span and the Hamilton scale	Compared to baseline, melatonin and clonazepam reduced sleep disorders in patients. The daytime sleepiness (ESS) was significantly increased in the clonazepam group. Patients treated with melatonin had better scores on the MMSE, five-word test, Hamilton scale at the end of the study period as compared with the clonazepam group. Changes in total point scores on the PSG at the end of week 6 were in favor of the group treated with melatonin	(197)
1 RBD patient	Case report	5 months	3 mg melatonin p.o./daily at bed time	Actigraphy, PSG	Significant reduction of motor activity during sleep, as measured by actigraphy. After 2 months' treatment, PSG showed no major changes except an increase of REM sleep	(198)
6 consecutive RBD patients	Open-label prospective case series	6 weeks	3 mg melatonin p.o./daily at bed time	PSG	Significant PSG improvement in 5 patients within a week which extended beyond the end of treatment for weeks or months	(199)
14 RBD patients	Open-label prospective case series	Variable	3–9 mg melatonin p.o./daily at bed time	PSG	Thirteen patients and their partners noticed a suppressing effect on problem sleep behaviors after melatonin administration. % tonic REM activity in PSG findings was decreased after melatonin administration. Melatonin concentrations in 10 RBD patients were under 30 pg/mL at maximal values, their mean 33.5 pg/mL RBD patients with low melatonin secretion tended to respond to melatonin therapy	(200)
14 RBD patients	Retrospective case series	14 months	3–12 mg melatonin p.o./daily at bed time	PSG	8 patients experienced continued benefit with melatonin beyond 12 months of therapy	(201)
45 RBD patients	Retrospective case series		All initially treated with clonazepam. When melatonin was used, it was given at a 10 mg p.o./daily at bed time		21 patients continued to take clonazepam, 8 used another medication, and 4 required a combination of medications to control symptoms adequately	(202)
25 RBD patients	Retrospective case series	27–53 months	6 mg melatonin p.o./daily at bed time		As compared to clonazepam-treated RBD patients (*n* = 18) patients receiving melatonin reported significantly reduced injuries and fewer adverse effects	(203)
8 RBD patients	Double blind, placebo-controlled trial	4 weeks	3 mg melatonin p.o./daily at bed time.	PSG	Reduced number of 30-s epochs of REM without atonia and reduced frequency of RBD episodes	(204)
1 RBD patient	Case report	5 years	2 mg prolonged release melatonin p.o./daily at bed time	PSG and DA transporter scintigraphy (DaTSCAN)	A then 72-year-old man was clinically suspected to suffer from PD in 2011. DaTSCAN revealed reduced DA transporter density and PSG confirmed the diagnosis of RBD. After 6 months of melatonin treatment, clinical signs of RBD were absent. Control PSG in 2014 confirmed normalized REM sleep with atonia. Additional DaTSCANs were performed in 2013 and 2015 indicated normalization of DA transporter density	(205)
4 RBD patients with concomitant obstructive sleep apnea	Open label	4 weeks	2 mg prolonged release melatonin p.o./daily at bed time	PSG	Treatment led to a relevant clinical improvement of RBD symptoms in all patients, so far untreated for the sleep related breathing disorder. REM without atonia incidence was high probably because of the untreated comorbid condition	(206)

Another consensus has claimed for trials with neuroprotective agents in RBD based on the high conversion rate from idiopathic RBD to PD (195). Indeed, the conversion rate to synucleinopathy in clonazepam-treated RBD patients is high (208, 209). Although no comparable data are available yet for melatonin-treated RBD patients, a recent observation by Kunz and Bes deserves to be considered (205). The investigators reported the increase in DA transporter density (as assessed by DA transporter scintigraphy) over successive years in a 72-year-old RBD male patient treated with 2 mg of slow release melatonin daily. After 6 months of gradual improvement, clinical and PSG signs of RBD disappeared. Whereas, the scan prior to melatonin treatment had clear signs of PD, the scan recorded 2 years later was considered borderline, with absence of any sign of PD 4 years after the first scan. The results were interpreted as a possible neuroprotective role for melatonin in synucleinopathy (205).

A phase advance in nocturnal melatonin secretion was reported in L-DOPA-treated parkinsonian patients (210, 211). L-DOPA-treated patients exhibited an increase in daytime melatonin secretion perhaps as an adaptive response to neurodegeneration (211). In a study aiming to examine circadian dysfunction as a cause for excessive sleepiness in PD, blunted circadian rhythms of melatonin were reported (212). The amplitude of the melatonin rhythm decreased in PD patients, mainly in those depicting excessive daytime sleepiness. Thus, a chronobiological approach to improve circadian function, such as timed exposure to melatonin and bright light, could serve as an adjuvant therapy for the non-motor manifestations of PD.

An association between motor fluctuations in PD and diurnal variation in circulating melatonin levels was postulated via possible interactions of melatonin with monoamines (DA, serotonin) in the striatal complex (213). Nearly half of the patients with PD showed L-DOPA-related motor complications after 5 years of treatment. In view of the results obtained in experimental parkinsonism discussed above, the use of melatonin as an adjuvant to decrease the therapeutic dosage of L-DOPA in PD deserves to be considered (214).

Wearing-off episodes in PD could be related to loss of the inhibitory motor effect of melatonin, since stimulation of globus pallidus improved motor symptoms and complications in patients with PD as well as inhibited the increase in daytime plasma melatonin levels found (215). Relevant to the subject of the present review, genetic susceptibility and life-style factors (e.g., smoking) have been entertained to explain the epidemiological that longer years of working night shifts are associated with reduced risk of PD and decreased melatonin levels (216).

Patients with PD showed decreased melatonin MT_1_ and MT_2_ receptor density in amygdala and substantia nigra (217). Supporting that a disrupted melatonergic system could be involved in the altered sleep/wake cycle seen in PD, an actigraphic study undertaken in 40 PD patients indicated that melatonin (50 mg/day at bedtime) increased nighttime sleep. Those patients taking 5 mg of melatonin only reported a significant improvement of subjectively evaluated sleep (196).

In another study, 18 PD patients were randomized after performing a basal PSG to receive melatonin (3 mg) or placebo 1 h before bedtime for 4 weeks (179). Although melatonin significantly improved the subjective quality of sleep, the motor dysfunction was not improved (179).

An important experimental study carried out in the MPTP monkey model of PD evaluated the effects of melatonin and L-DOPA on sleep disorders as monitored by PSG (218). The combined treatment of melatonin and L-DOPA significantly curtailed sleep fragmentation at night and sleep episodes during the day seen in MPTP-treated monkeys, thus indicating that melatonin treatment may have the therapeutic potential to treat sleep disorder in PD patients.

Exposure to 1–1.5 h of light (1,000–1,500 lux) prior to bedtime reduced bradykinesia and rigidity in PD patients, as well as agitation and psychiatric side effects (219). The authors concluded that suppressing melatonin secretion with bright light may have a therapeutic value for treating the symptoms of PD (220). However, suppression of melatonin secretion may not be the likely mechanism by which artificial light exerts its therapeutic effect, as shown in depressive patients subjected to phototherapy (221). In any event, the circadian system is considered a novel diagnostic and therapeutic target in PD (212, 222).

**Figure 1:**
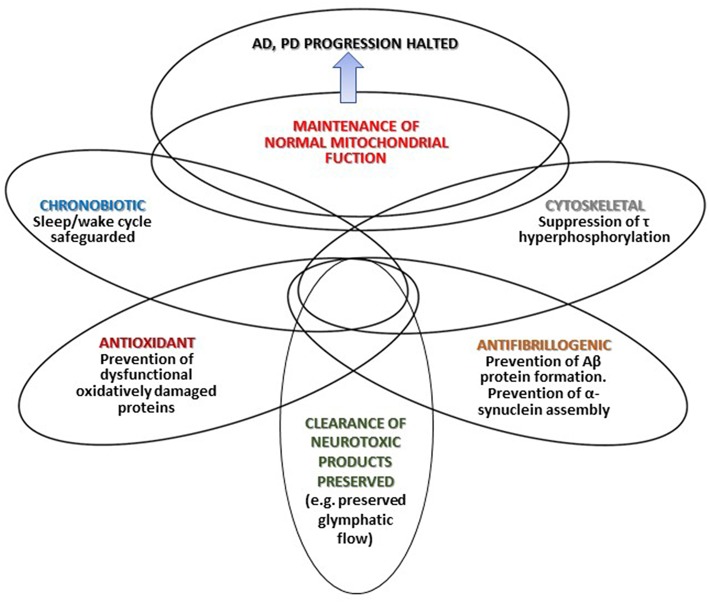
The different mechanisms through which melatonin may halt AD and PD progression.

Figure 1 summarizes that different mechanisms by which melatonin may halt AD and PD progression. Depicted intersections in the Figure represent the multiple effects of melatonin and the different degree of overlap (interrelations and mutual influences) discussed in the text.

## Conclusions

Because of both hypnotic and chronobiotic properties, the use of melatonin has been recommended for treatment of insomnia (223, 224). Several meta-analyses support such therapeutic role (225–227). A consensus of the British Association for Psychopharmacology on evidence-based treatment of insomnia concluded that melatonin is the first choice treatment when a hypnotic is indicated in patients over 55 years (228).

As discussed in this article, studies using 2–5 mg melatonin/day are unsuitable to give appropriate comparison with data on neurodegeneration protection derived from animal studies. Melatonin is remarkably atoxic and its safety is very high. Lethal dose 50 (LD50) for intraperitoneal melatonin injection was 1,131 mg/kg for mice and 1,168 mg/kg for rats. However, LD50 could not be constructed after oral administration of up to 3,200 mg of melatonin/kg in rats, or after subcutaneous injection of up to 1,600 mg/kg in rats and mice (229). In humans, melatonin has a high safety profile and it is usually remarkably well-tolerated (Table 6). Presently, the only option for the incumbent physician interested in the use of melatonin as a cytoprotective is the off-label indication of the drug.

**Table 6:** Safety for off label prescription of melatonin.

**Clinical condition**	**Melatonin dose**	**References**
Dermal hyperpigmentation	1 g/day p.o. for 1 month	(230)
Parkinson's disease	0.25 and 1.25 mg/kg i.v.	(231)
Amyotrophic lateral sclerosis	60 mg/day p.o. for 13 months	(232)
Amyotrophic lateral sclerosis	300 mg/day, rectal for 2 years	(233)
Muscular dystrophy	70 mg/day for 9 months	(234)
Multiple sclerosis	50–300 mg/day p.o. for 4 years	(235)
Liver surgery	50 mg/kg	(236)
Healthy individuals	80 mg/h for 4 h	(237)
Healthy women	300 mg/day for 4 months	(238)
Dose escalation in healthy individuals	10–100 mg p.o.	(239)
Dose escalation in healthy individuals	10–100 mg p.o.	(240)

Off-label drugs are defined as drug uses that not included in the indications or dosage regimens listed by the administrative body that registers, controls, and monitors medicines authorized, e.g., the Food and Drug Administration in USA (241). Off label drug use is common in intensive care unit, pediatrics, psychiatry and oncology (242–245). In general, no law prohibits off-label drug use and prescribing off-label is legally accepted in most legislations (246).

In Argentina, the National Administration for Medicaments, Food and Medical Technology (ANMAT) approved melatonin (3 mg capsules or tablets) as an over-the-counter medication in 1995. In 2017 ANMAT authorized a prolonged release preparation of 2 mg melatonin (Circadin^R^) as a prescription drug. Although ANMAT cannot authorize the use of a medication for an indication not listed in the package leaflet, it does not mean that the indication of a medication for other clinical situations is prohibited. In accordance to ANMAT, The off-label prescriptions are “the sole responsibility of the attending physician, who performs them in the full exercise of their professional activity, based on their experience and the available scientific knowledge, motivated by the need to provide an answer to health problems for which there are no standards of treatment or that, in case of existing, they are very difficult to access.”

In many countries, melatonin is used as a food supplement or dietetic products. Indeed, the European Food Safety Authority (EFSA) has endorsed the health claim that melatonin reduces sleep onset latency (247, 248). Thus, melatonin, melatonin-rich food and bioextracts could now be developed.

Overexpression of melatonin in plants facilitates the germination of seeds and protects plants from abiotic and biotic stress (249–251) and potentially genetically manipulated plants may have use in human nutrition. In parallel, toxicity of long-term melatonin use must be evaluated.

In conclusion, from animal studies several potentially useful effects of melatonin, like those in neurodegenerative disorders, need high doses of melatonin to become apparent. Regardless of the amount of experimental data gathered as far as how melatonin acts in animal and cell models, in most cases it is not known whether it works as a chronobiotic drug, as an endogenous antioxidant or as an immunomodulatory compound. This is an important caveat deserving consideration (252).

Although melatonin is remarkably atoxic and its safety is very high in adults, caution must be exerted with melatonin use in children taking in consideration that melatonin is known to inhibit LH secretion from neonatal pituitary gonadotrophs in the rat (253). Even if similar effect has not yet been documented in human, it cannot be excluded that treatment with high doses of exogenous melatonin could influence the development of the reproductive system.

## Author Contributions

The author confirms being the sole contributor of this work and has approved it for publication.

### Conflict of Interest Statement

The author declares that the research was conducted in the absence of any commercial or financial relationships that could be construed as a potential conflict of interest.
